# Posterior Reversible Encephalopathy Syndrome (PRES): Pathophysiology and Neuro-Imaging

**DOI:** 10.3389/fneur.2020.00463

**Published:** 2020-06-16

**Authors:** Redmond-Craig Anderson, Vishal Patel, Nasim Sheikh-Bahaei, Chia Shang J. Liu, Anandh G. Rajamohan, Mark S. Shiroishi, Paul E. Kim, John L. Go, Alexander Lerner, Jay Acharya

**Affiliations:** Department of Radiology, Keck School of Medicine, University of Southern California, Los Angeles, CA, United States

**Keywords:** PRES (posterior reversible encephalopathy syndrome), neuroimaging, neuroradiology, pathophysiology, cerebrovascular abnormalities

## Abstract

Posterior reversible encephalopathy syndrome (PRES) represents a unique clinical entity with non-specific clinical symptoms and unique neuroradiological findings. This syndrome may present with a broad range of clinical symptoms from headache and visual disturbances to seizure and altered mentation. Typical imaging findings include posterior-circulation predominant vasogenic edema. Although there are many well-documented diseases associated with PRES, the exact pathophysiologic mechanism has yet to be fully elucidated. Generally accepted theories revolve around disruption of the blood-brain barrier secondary to elevated intracranial pressures or endothelial injury. In this article, we will review the clinical, typical, and atypical radiological features of PRES, as well as the most common theories behind the pathophysiology of PRES. Additionally, we will discuss some of the treatment strategies for PRES related to the underlying disease state.

## Introduction

Posterior reversible encephalopathy syndrome (PRES), first described by Hinchey et al. in 1996, represents a neurological disorder with varied clinical presentation and typical imaging findings of parieto-occipital predominant pattern of vasogenic edema (1, 2). There are numerous documented causes of PRES, with cases first described in the setting of elevated arterial pressures. Examples of clinical scenarios in which PRES may be seen include: hypertensive emergency, (pre)eclampsia, renal disease, autoimmune disorders, and cytotoxic medications, among others (3, 4) (Table 1). PRES can occur in any age group and has a higher occurrence rate in female patients (7, 8). Although current literature is relatively sparse compared to adult populations, particular mention should be made of PRES in the pediatric patient. Pediatric patients have a similar clinical presentation as the adult population, with hypertension, seizure, and altered mental status being common disease manifestations (9). Despite most cases of pediatric PRES being reported in oncology patients, especially the post-stem-cell transplant patients (10, 11), a study by Gupta et al. (12) found that renal disease was perhaps the most common cause of PRES in the pediatric patient. In their study, pediatric patients tended to have more atypical imaging findings (62.5%), including frontal lobe involvement (56%).

**Table 1:** Major disease states associated with PRES.

**Hypertensive diseases**	**Endothelial dysfunction**
Hypertension (primary or secondary causes)	Cytotoxic substances: chemotherapy, immunosuppressants, etc. (5, 6). •Bevacizumab •Carboplatin •Cisplatin •Cyclosporin •Cytarabine •Docetaxel •Irinotecan •Methotrexate •Oxaliplatin •Paclitaxel •Prednisone •Rituximab •Vincristine
Renal disease	Infection (sepsis)
Autoimmune disorders	(Pre)eclampsia
	Autoimmune disorders

Clinical manifestations are acute to subacute and range from headache and visual disturbances to altered levels of consciousness and seizure in more severe cases (1). Treatment is generally aimed at targeting the underlying cause, with generally reversible symptoms and imaging findings in most cases (8). Although outcomes are generally favorable with proper management, poor clinical outcomes have been associated with pre-existing diabetes mellitus, and involvement of the corpus callosum; however, other reliable imaging biomarkers for prognostication are currently lacking (13). Neuroradiological imaging plays a fundamental role in the diagnosis of PRES with the typical imaging features best appreciated on magnetic resonance imaging (MRI) (2).

## Pathophysiology

**Figure 1:**
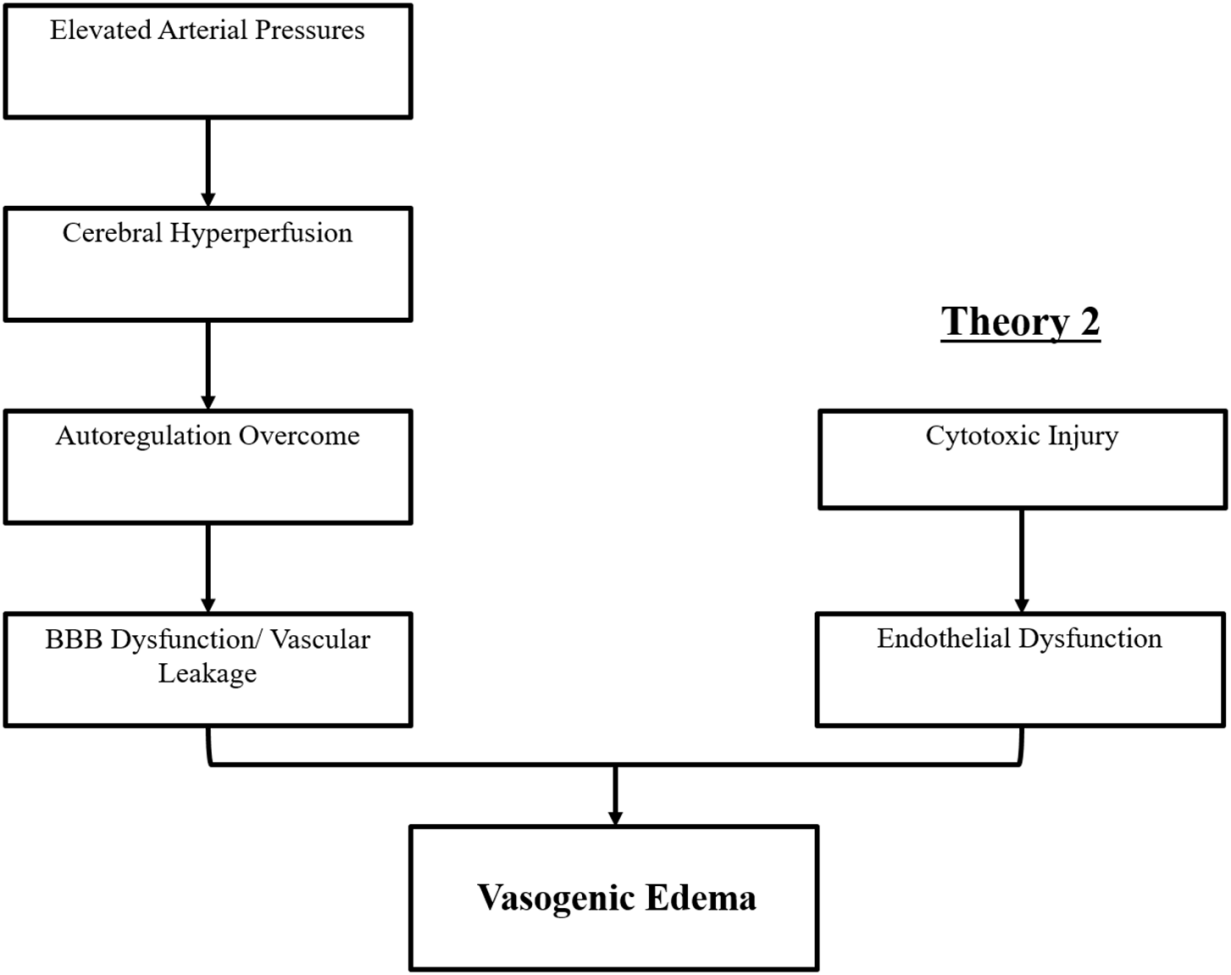
Two major theories of the pathophysiology of PRES. Theory 1 is the hypertensive and cerebral hyperperfusion theory and Theory 2 is the endothelial dysfunction theory.

The precise pathophysiologic mechanism(s) behind PRES have yet to be fully elucidated and remain controversial (3). There are currently two major proposed mechanisms for the pathophysiology of PRES (Figure 1). The first theory proposes increased arterial pressures as the primary factor (8). Rapid rises in blood pressures eventually overcome the autoregulatory capabilities of the cerebral vasculature causing vascular leakage and resultant vasogenic edema (14). There is eventual blood-brain barrier (BBB) dysfunction with proteins passing through the tight-junction (15). The areas supplied by the posterior circulation (vertebral arteries, basilar artery, and posterior cerebral arteries) are at exceptional risk compared to the anterior circulation (internal carotid arteries, middle cerebral arteries, and anterior cerebral arteries) due to the lack of sympathetic tone of the basilar artery vasculature (8). A related theory proposed by some postulates that extreme hypertension results in vasospasm and local ischemia which causes BBB breakdown and resultant vasogenic edema, as was observed in patients being treated with immunosuppressive agents cyclosporin A and FK-506 (16). The disruption in the BBB causes the typical findings of vasogenic edema vs. cytotoxic edema (which may be seen in the setting of acute infarct and represents increased intracellular water content due to loss of the usual osmotic gradient in the setting of cell death (17).

**Figure 2:**
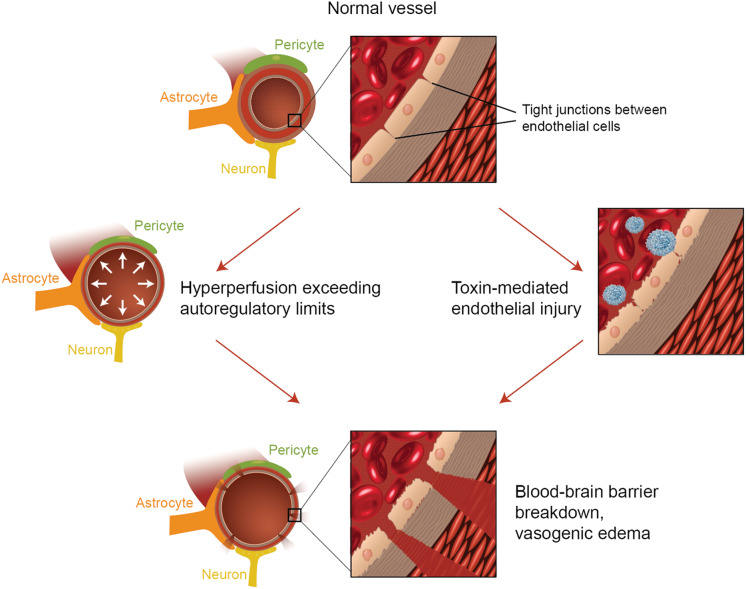
Illustration of the two major theories of the underlying pathophysiology of PRES. Acknowledgment: Courtesy of Caroline O'Driscoll, MA (She illustrated this on her own and is an employee of our university).

The second major theory addresses the fact that up to 30% of patients with PRES do not exhibit the elevated arterial pressures necessary to exceed the autoregulatory control of the cerebral vasculature (18, 19). This theory proposes that endothelial dysfunction is the primary culprit, which may be caused by various endogenous or exogenous toxins (20). This theory can explain the findings of PRES seen in patients receiving immunosuppressive medications and/or chemotherapy and also those patients with sepsis (21, 22). In this model, circulating toxins cause vascular injury with resultant development of vasogenic edema. The endothelial damage causes further release of vasoconstrictive and immunogenic agents, which may cause vasospasm and/or increased vascular permeability. Ultimately, endothelial dysfunction allowing for vascular leakage and vasogenic edema is the driving factor behind PRES, regardless of the primary cerebral vasculature abnormality (in the case of arterial hypertension) or secondary to circulating toxins. A summary of these two theories as well as a list of previously reported chemotherapeutics and other immunosuppressants is shown in Figure 2.

Clinical manifestations depend on the involved region(s) of the brain; thus, the presentation may be broad. For example, primary involvement of the occipital lobes may result in visual disturbances/hallucinations. Focal neurological deficits corresponding to the location of focal lesions occurs in ~5–15% of patients with PRES (23). Rarely, spinal cord involvement may result in clinical signs and symptoms of myelopathy or paralysis (24).

## Imaging

**Figure 3:**
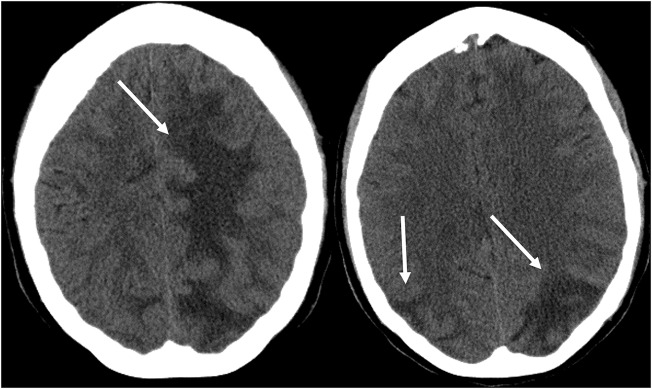
Patient with systemic lupus erythematosus and rapidly progressive glomerulonephritis presenting with seizure. Non-contrast CT images of the head demonstrates vasogenic edema in the bilateral parietal and occipital lobes, left greater than right, as well as extension into the left frontal lobe.

**Figure 4:**
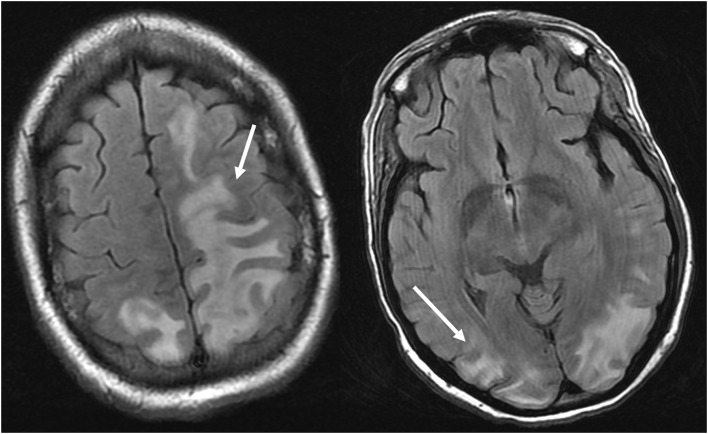
Patient with systemic lupus erythematosus and rapidly progressive glomerulonephritis presenting with seizure. T2-FLAIR images of the head demonstrates vasogenic edema in the bilateral parietal and occipital lobes, left greater than right, as well as extension into the left frontal lobe. Note that with the vasogenic pattern of edema, there is sparing of signal abnormality in the cortex.

**Figure 5:**
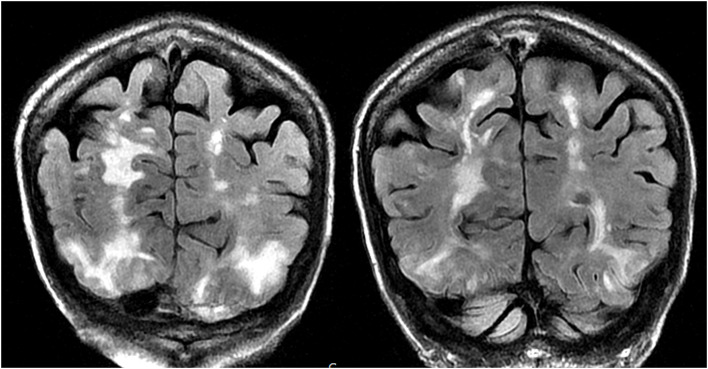
Patient with a history of primary myelofibrosis and bone marrow transplant on Tacrolimus presenting with first time seizure. Coronal T2-FLAIR sequences demonstrate extensive signal abnormality in the bilateral occipital and parietal lobes, as is typical with PRES.

As its name suggests, PRES typically manifests on imaging studies as posterior-predominant white matter vasogenic edema. The parietal and occipital lobes are almost universally involved and findings are typically symmetrical and bilateral (1). Involvement of the frontal lobes, particularly adjacent to the superior frontal sulci, is also commonly seen. Vasogenic edema, although it can involve the cerebral gray matter, is often more readily appreciated in the subcortical white matter. CT examination is often the initial imaging test in setting of acute neurological symptoms and may demonstrate white matter hypoattenuation in affected regions (25) (Figure 3: CT of PRES). Overall, findings are best depicted by MRI which exhibits increased sensitivity and better anatomical characterization compared to CT (26). Additionally, MRI may help to distinguish other pathological states that may manifest clinically similarly to PRES. The T2-weighted and T2 FLAIR (fluid-attenuated inversion recovery) sequences, in particular, are most useful to detect vasogenic edema on MRI (Figure 4: MR of PRES; Figure 5: MR of PRES Coronal).

The differential diagnosis for PRES is broad and includes entities with similar confluent T2 white matter hyperintensity. Examples include: ischemia/infarction (particularly posterior circulation), demyelinating diseases, infectious etiologies (meningitis, encephalitis), progressive multifocal leukoencephalopathy (PML), vasculitis, and various metabolic disorders (27). A clinically related entity called reversible cerebral vasoconstriction syndrome (RCVS) is thought to be caused by alterations in cerebral vascular tone resulting in vasoconstriction. RCVS manifests as recurrent thunderclap headaches, seizure, stroke, and non-aneurysmal subarachnoid hemorrhage (28), which could be mistaken for PRES on a clinical basis. This entity typically occurs in the post-partum period or after exposure to adrenergic or serotonergic medications. RCVS can typically be diagnosed with angiographic studies demonstrating multifocal areas of narrowing involving the cerebral arteries (29). This diagnosis can be confounded with the fact that RCVS and PRES often occur concomitantly, which the neuroradiologist should be aware of to avoid misdiagnosis (30).

PRES can typically be distinguished from acute ischemia because the latter invariably demonstrates cytotoxic edema and diffusion restriction. Restricted diffusion in acute ischemia can be easily detected on diffusion-weighted imaging (DWI) and apparent diffusion coefficient (ADC) as a hyperintense signal on DWI with corresponding decreased signal on ADC (due to the relatively decreased movement of intracellular water molecules). Vasogenic edema in the setting of PRES, on the other hand, may show hyperintense signal on DWI that is not accompanied by a corresponding decreased signal on ADC (31). Additionally, acute ischemia tends to be unilateral and within a singular vascular territory. While assessing for diffusion restriction to differentiate PRES from ischemic abnormality generally is reliable, there are rare cases of PRES that may be associated with areas of diffusion restriction superimposed upon areas of the more classically seen isolated vasogenic edema.

“Advanced” imaging techniques in PRES have recently been described as an adjunct tool in difficult or equivocal cases. These advanced imaging techniques include: CT/MR perfusion, MR Spectroscopy (MRS), Susceptibility weighted imaging (SWI), and nuclear medicine techniques, including single-photon emission tomography (SPECT) and positron emission tomography (PET) with varying radiotracers. Although a full discussion is beyond the scope of this review, a variety of imaging findings can be seen on these advanced techniques to help suggest a diagnosis of PRES. Hyperperfusion may be seen on CT/MR perfusion studies demonstrated by increased cerebral blood flow and blood volume with reduced time to perfusion and mean transit time (32) although cases of hypoperfusion have been reported (33). On MRS, there is generally a reduction in the N-Acetylaspartate (NAA)/Creatine (Cr) and NAA/Choline (Chol) ratio, suggestive of a disruption of normal synapses and neuroaxonal function (34). SWI can help to identify the presence of hemorrhage in PRES, with higher sensitivity than GRE imaging (35). SPECT/PET imaging typically demonstrates either hyperperfusion or hypoperfusion (similar to CT/MR perfusion studies) with low metabolism by FDG-PET (36).

Additionally, PRES can be distinguished from other conditions such as autoimmune encephalitis in the setting of acute disseminated encephalomyelitis (ADEM) by the former's diffuse bilateral but asymmetric vasogenic edema (37). PML may have a similar appearance when compared to PRES, having a parieto-occipital predominance, but may be distinguished by its more unilateral or asymmetric involvement, as well as predilection for subcortical white matter (38).

## Atypical Imaging Features

**Figure 6:**
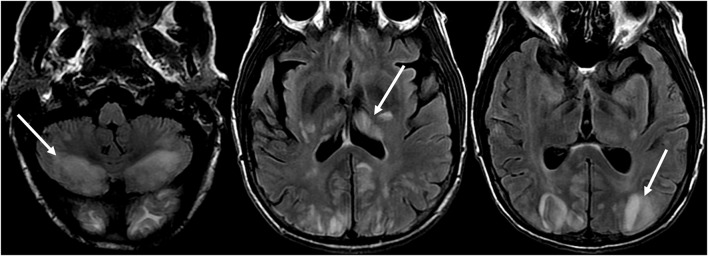
Patient with liver transplantation 6 weeks earlier. The patient was started on Tacrolimus after liver transplantation. T2-FLAIR images of the brain demonstrate signal abnormality in the occipital lobes. There is also extensive signal abnormality seen in the bilateral cerebellar hemispheres and within the thalami. These findings quickly resolved after stopping the Tacrolimus.

**Figure 7:**
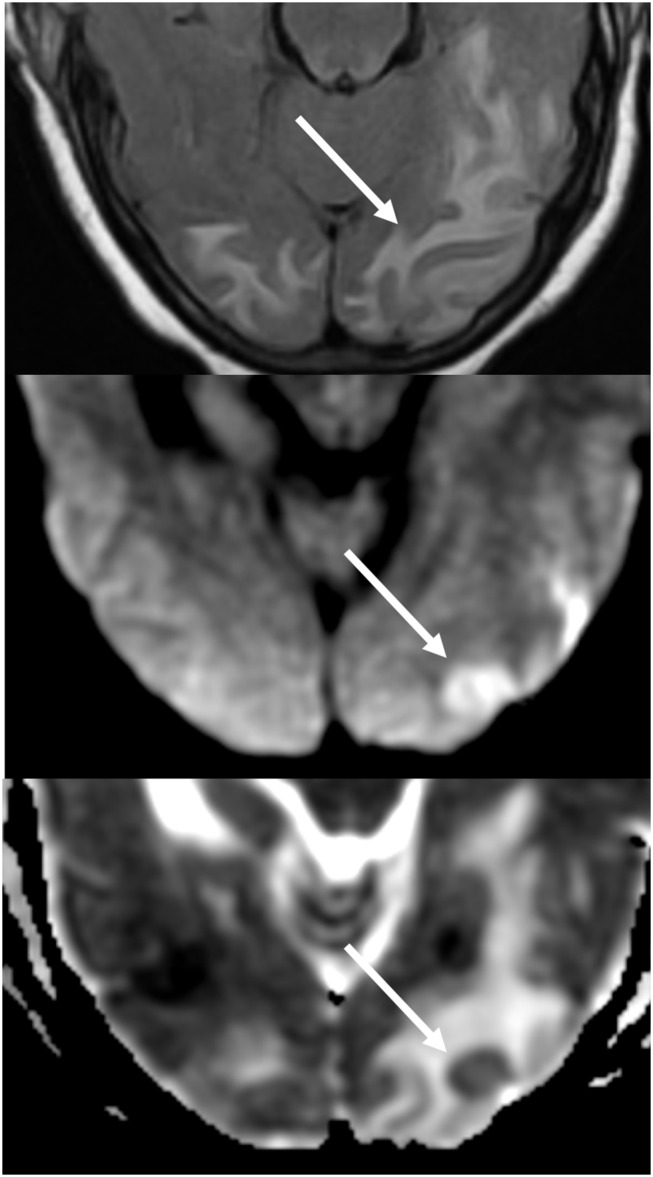
Patient with uncontrolled hypertension presenting with alteration of mental status. T2-FLAIR (top) image demonstrates edema in the occipital lobes. DWI (middle) and ADC map (bottom) images demonstrates a small arrow of restricted diffusion, with hyperintense signal on DWI and corresponding hypointensity on the ADC map.

Atypical features of PRES include areas of contrast enhancement, hemorrhage, or diffusion restriction (39). Although the parietal and occipital lobes are generally involved, atypical areas of involvement may be seen, including: brainstem, cerebellum, corpus callosum, and other cerebral areas, with more common areas including the frontal lobes (seen in up to 68%) and inferior temporal lobes (up to 40%) (23, 40) (Figure 6: Cerebellum; Figure 7: Brainstem).

**Figure 8:**
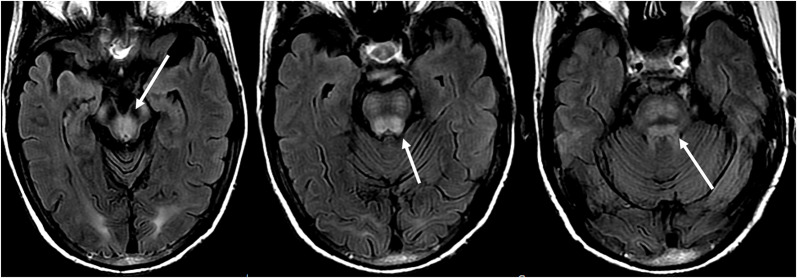
Patient with a history of liver transplantation two weeks earlier on Tacrolimus. T2-FLAIR images show signal abnormality within the midbrain, pons, and superior cerebellar peduncles.

**Figure 9:**
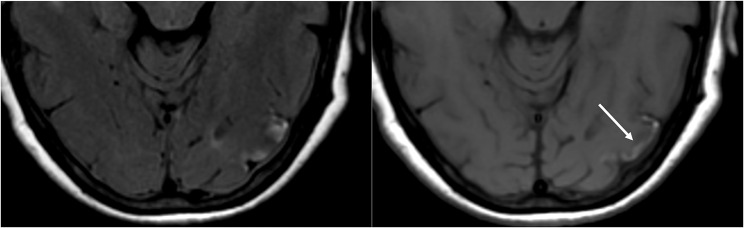
T2-FLAIR (left) image in a patient with uncontrolled hypertension and prior imaging indicating PRES (see Figure 6), now controlled and 6 weeks later, demonstrates resolution of the previously seen edema. Small areas of gliosis due to injury are seen in the left temporal lobe. Axial non-contrast T1 shows curvilinear cortical laminar necrosis related to the prior injury related to PRES. While PRES generally is fully reversible, it may result in permanent injury in some situations.

**Figure 10:**
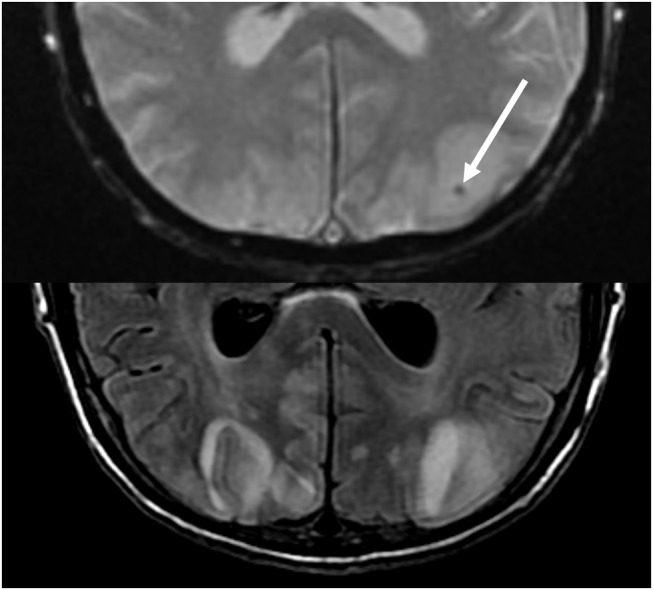
Patient with a prior liver transplantation on Tacrolimus. T2 gradient recalled echo (top) demonstrates a focal area of hemorrhage within the vasogenic edema in the left occipital lobe. T2-FLAIR demonstrates the more typical finding related to PRES with signal abnormality in the bilateral occipital lobes.

Additionally, an early finding of PRES, which may precede the typical parieto-occipital edema includes mild sulcal FLAIR hyperintensity and leptomeningeal enhancement on post-contrast T1 weighted images, as described by Nakagawa et al. (41). Benziada-Boudour et al. (42) described a concurrent development of cytotoxic edema, resulting in restricted diffusion (Figure 8: Diffusion Restriction). Inherent in the name of the disease process, the findings related to PRES are usually reversible, with normalization of clinical and imaging findings once the inciting issue is treated. However, in some cases, areas of restricted diffusion can ultimately result in permanent injury to the brain parenchyma (Figure 9: Laminar Necrosis). Hemorrhage is less commonly seen in PRES, occurring in 5–30% of cases, but should be recognized as to not mistake this finding for another pathological entity in the appropriate clinical setting of PRES (39). Imaging findings in hemorrhage may include: focal hematoma, petechial gyral hemorrhage, and/or subarachnoid hemorrhage (43) (Figure 10: Hemorrhage).

## Treatment

**Figure 11:**
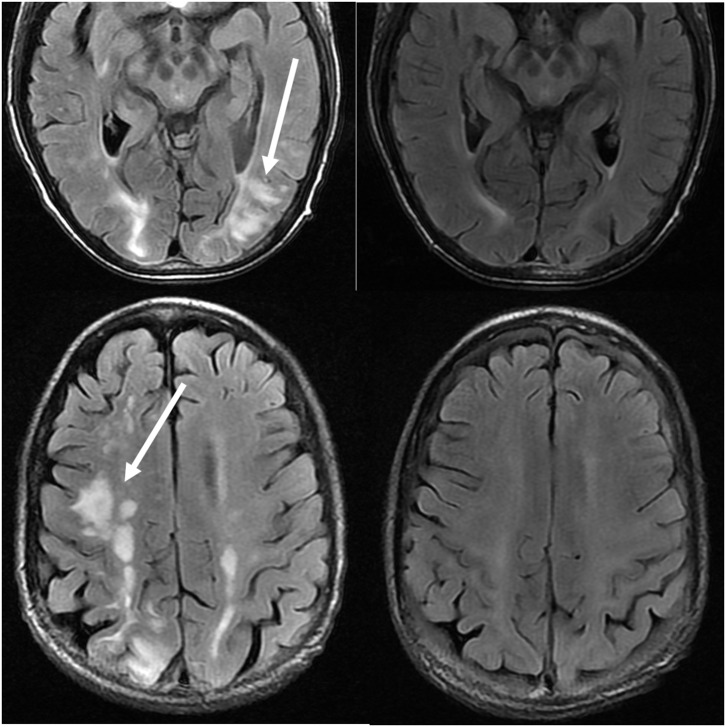
Patient with a history of primary myelofibrosis and bone marrow transplant on Tacrolimus. Axial T2-FLAIR images demonstrate the areas of signal abnormality in the parietal and occipital lobes, and right frontal lobe (left images). The Tacrolimus was stopped and the follow up images (right images) were obtained 6 weeks after the initial images.

Treatment of PRES is typically aimed at controlling the primary etiology causing PRES (44). For example, in cases of elevated arterial pressures, treatment is aimed at correcting the elevated blood pressures in a controlled environment, similar to the approach for hypertensive urgency/ emergency (45). Typically, a non-rapid reduction in blood pressure is sought to avoid the risk of causing ischemic cerebral disease as a result of drastic blood pressure lowering (46). Occasionally, anticonvulsant medications are used as adjunct therapy, although the optimal agent(s), timing, and length of treatment remain controversial (4) (Figure 11: Before and After).

**Figure 12:**
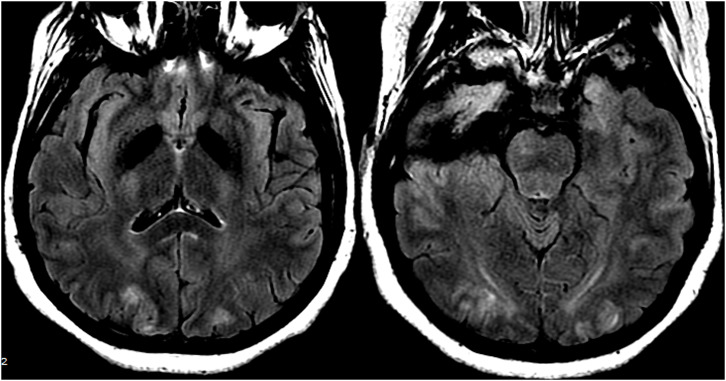
Patient with eclampsia and presenting with seizure. Axial T2-FLAIR images demonstrate symmetric signal abnormality in the bilateral occipital lobes.

In cases of (pre)eclampsia, treatment is aimed at the timely delivery of the fetus as well as blood pressure management and magnesium sulfate for seizure prophylaxis (47). In the setting of PRES induced by chemotherapeutic or other immunosuppression agents, tapering or absolute discontinuation of the drug has shown both clinical and radiological improvement (48) (Figure 12: Eclampsia). Hypomagnesemia is a common finding in PRES and a possible etiological factor. Hence, authors have suggested that magnesium supplementation may be a useful adjunct in PRES management (49).

## Conclusion

PRES is a unique entity with characteristic clinical and neuroradiological findings, in addition to myriad well-documented causes. Although the precise pathophysiologic mechanism(s) behind PRES has yet to be elucidated (and indeed may be due to a combination of interrelated processes), the generally accepted mechanism is dysfunction of the blood-brain barrier resulting in vasogenic edema with posterior-circulation predominance. Imaging features are best evaluated on fluid-sensitive MR sequences which reveal parieto-occipital predominant white matter T2 hyperintensities, although many atypical imaging features can be seen and should be kept in mind when evaluating challenging cases. Various advanced imaging tools are available to help in difficult or equivocal cases. Treatment is aimed at managing the underlying cause with specific attention to blood pressure monitoring and possible seizure prophylaxis.

## Author Contributions

R-CA and JA: Contributions to the manuscript, figures, and tables. VP: Contributions to the manuscript and figures. NS-B, CL, AR, MS, PK, JG, and AL: Contributions to the manuscript.

## Conflict of Interest

The authors declare that the research was conducted in the absence of any commercial or financial relationships that could be construed as a potential conflict of interest.
